# Botulinum Toxin for First Bite Syndrome: A Symptom‐Guided “Follow‐The‐Pain” Injection Strategy

**DOI:** 10.1002/hed.70378

**Published:** 2026-07-01

**Authors:** Rosario Marchese‐Ragona, Gianmarco Giunta, Daniela Adamo, Elisabetta Zanoletti, Daniele Manfredini, Domenico Antonio Restivo

**Affiliations:** ^1^ Section of Otorhinolaryngology—Head and Neck Surgery, Department of Neuroscience (DNS) University of Padova Padova Italy; ^2^ Department of Life Science, Health, and Health Professions Link Campus University Rome Italy; ^3^ Department of Biomechanical Diseases, School of Dentistry University of Siena Siena Italy; ^4^ Department of Clinical and Experimental Medicine, Physical Medicine and Rehabilitation Unit University of Messina Messina Italy

**Keywords:** botulinum toxin type a, First Bite Syndrome, head and neck surgery, pain, postoperative complication

## Abstract

**Background:**

To evaluate botulinum toxin type A (BoNT‐A) for First Bite Syndrome (FBS) and assess a symptom‐guided “Follow‐the‐Pain” (FTP) injection strategy in patients with incomplete response to standard treatment.

**Methods:**

Consecutive patients with postoperative FBS were treated with ultrasound‐guided intraparotid BoNT‐A injection. Patients with partial or poor response received additional targeted injections (15–25 U) at patient‐reported sites of maximal pain using the FTP protocol. Pain intensity was assessed using a Visual Analog Scale (VAS), and outcomes were monitored with a biweekly patient diary.

**Results:**

Five patients (mean age, 41 years) were included. Standard treatment resulted in marked improvement in one patient, partial benefit in three, and no response in one. The four patients with persistent symptoms subsequently underwent the FTP protocol, achieving additional pain reduction. Symptom recurrence generally required repeat treatment at 5–6‐month intervals.

**Conclusions:**

BoNT‐A appears effective for FBS. In patients with persistent or partially controlled symptoms, a symptom‐guided FTP strategy may provide additional benefit within a stepwise management approach.

**Level of Evidence:**

4.

**Abbreviations**
BoNT‐A: botulinum toxin type A
CGRP: calcitonin gene–related peptide
FBS: First Bite Syndrome
FTP: “follow‐the‐pain”
ICHD: International Classification of Headache Disorders
NRS: Numeric Rating Scale
OHIP‐14: oral health impact profile
PGIC: patient global impression of change
PREEMPT: Phase III REsearch Evaluating Migraine Prophylaxis Therapy
TRPV1: transient receptor potential vanilloid 1
VAS: Visual Analog Scale

## 1 Introduction

First Bite Syndrome (FBS) [1] is an uncommon but increasingly recognized postoperative pain condition, first systematically described by Netterville et al. in 1998 [2] in patients undergoing paraganglioma surgery. It is characterized by sudden, severe pain localized to the ipsilateral parotid or preauricular region, occurring with the first bite of a meal and rapidly diminishing with continued mastication. Pain is typically described as sharp, cramping, or electric shock–like and may radiate toward the ear or jaw. In some patients, symptoms can also be triggered by salivation or anticipatory gustatory stimuli even before food intake. Despite its brief duration during each episode, the repetitive occurrence of FBS may significantly impair quality of life, leading to food avoidance, weight loss, and psychological distress. Originally described as a complication of parapharyngeal space surgery [3], FBS has since been associated with deep lobe parotidectomy [4], carotid body tumor resection, procedures involving the auriculotemporal nerve, cervical lymph node dissection, and operations in proximity to the cervical sympathetic chain or infratemporal fossa. High‐risk procedures—particularly those involving the parapharyngeal space, deep lobe parotidectomy, and sympathetic chain surgery—carry a reported incidence ranging from 9% to 20%, as documented by Linkov et al. [5] Although most cases occur following surgery, rare idiopathic presentations or cases associated with underlying head and neck tumors have also been described [6].

The pathophysiology of FBS is most explained by the autonomic imbalance theory. The parotid gland receives dual autonomic innervation: parasympathetic fibers originating from the glossopharyngeal nerve stimulate watery salivary secretion via the auriculotemporal nerve, while sympathetic fibers arising from the superior cervical ganglion regulate vasomotor tone and influence myoepithelial contractility [7]. Surgical injury to the sympathetic pathway results in denervation hypersensitivity of parotid myoepithelial cells, a phenomenon consistent with Cannon's law. Consequently, unopposed parasympathetic stimulation during gustatory activation leads to exaggerated myoepithelial contraction and acute parotid pain at the onset of mastication. The rapid attenuation of symptoms with continued mastication likely reflects receptor desensitization or functional fatigue of the contractile response [3, 4, 5, 6, 7].

Diagnosis of FBS is primarily clinical and relies on recognition of its characteristic features: unilateral parotid pain triggered by the first bite of a meal and rapid attenuation of symptoms with continued chewing. No specific imaging or laboratory markers currently exist, and awareness of surgical history and autonomic anatomy is essential to distinguish FBS from other causes of facial pain such as trigeminal neuralgia, temporomandibular disorders, or persistent postoperative neuropathic pain [3, 4, 5, 6, 7, 8].

Management of FBS remains challenging. Conventional analgesics and oral pharmacologic therapies often provide limited benefit. In 2008, Ali et al. first reported the use of botulinum toxin type A (BoNT‐A) for FBS [9], describing a case of severe, refractory symptoms treated with ultrasound‐guided injection of 75 units of BoNT‐A into the parotid gland with particular attention to regions of maximal pain, resulting in significant clinical improvement. Subsequent case reports and small series have further supported the efficacy of intraparotid BoNT‐A injections in reducing first‐bite pain intensity [10, 11, 12]. Proposed mechanisms include inhibition of acetylcholine release and modulation of peripheral nociceptive pathways through suppression of pain‐related mediators such as calcitonin gene–related peptide (CGRP) and glutamate, as well as down‐regulation of TRPV1 receptor activity. In addition to its effects on cholinergic transmission, BoNT‐A may therefore exert peripheral antinociceptive activity by modulating sensory neurotransmission, supporting its potential role in the management of focal postoperative pain conditions [13].

Despite these proposed mechanisms and encouraging clinical reports, significant heterogeneity persists in injection techniques, dosing strategies, and anatomical targeting. A systematic review by Shaikh et al. [14] highlighted the predominance of small case series and the lack of consensus regarding optimal injection protocols, particularly in patients with incomplete or absent response to initial treatment. Currently, no structured escalation strategy has been clearly defined for refractory cases. The present study aims to evaluate the clinical effectiveness of ultrasound‐guided BoNT‐A injection in patients with postoperative FBS and to introduce a symptom‐guided injection strategy, termed the “Follow‐the‐Pain” (FTP) technique, inspired by the paradigm originally described by Blumenfeld et al. [15] in the context of botulinum toxin treatment for migraine and tension‐type headache. This approach is proposed as a rational escalation strategy for patients with persistent or incomplete response following standard intraparotid injection.

## 2 Materials and Methods

This retrospective case series included all consecutive patients diagnosed with FBS following head and neck surgery who were treated with BoNT‐A at the Neurolaryngology Outpatient Clinic, Department of Otolaryngology–Head and Neck Surgery, University of Padua, between January 2021 and May 2026.

Patients were eligible if they met the following inclusion criteria: (1) clinical diagnosis of FBS characterized by parotid or preauricular pain consistently triggered by the first bite of a meal; (2) history of previous head and neck surgery involving the parotid gland, parapharyngeal space, cervical region, or carotid body; (3) persistence of symptoms for at least 3 months postoperatively.

Exclusion criteria included alternative causes of facial pain, such as trigeminal neuralgia, temporomandibular joint disorders, active infection, or tumor recurrence.

The study was conducted in accordance with the guidelines of the Declaration of Helsinki and the principles of Good Clinical Practice. According to institutional policies for studies involving anonymized clinical data, formal ethics committee approval was not required. All patients provided consent for the use of their anonymized clinical data for research and educational purposes.

### 2.1 Standard Injection Protocol

All injections were performed by the same senior otolaryngologist with extensive experience in ultrasound‐guided salivary gland procedures, ensuring procedural consistency across the entire cohort. All patients initially underwent treatment using a standard intraparotid injection protocol. The dosing strategy was defined on the basis of previously published case series reporting effective doses ranging from 40 to 75 units for intraparotid BoNT‐A injection in FBS [9, 10, 11, 12]. A total dose of 40–50 units was selected as a conservative and well‐tolerated starting dose, consistent with the lower range reported in the literature and with the institutional experience in BoNT‐A treatment of salivary and autonomic disorders of the head and neck [8, 16, 17]. BoNT‐A (Xeomin, incobotulinumtoxinA) was reconstituted with 2.0 mL of unpreserved 0.9% saline per vial, yielding a final concentration of 50 U/mL. The toxin was administered under real‐time ultrasound guidance into the affected parotid gland, with a total dose of 40–50 units divided into 8–10 injection points of 5 units each, evenly distributed throughout the gland parenchyma to ensure uniform toxin diffusion. This distribution pattern was adopted to maximize coverage of the glandular tissue while minimizing the risk of focal over‐injection and adjacent structure involvement. In the patient who had previously undergone parotidectomy, BoNT‐A was injected into the subcutaneous tissue of the parotid region corresponding to the surgical bed, using the same dose and distribution strategy.

### 2.2 Pain‐Oriented “Follow‐the‐Pain” (FTP) Protocol

In patients who showed minimal or incomplete clinical response after standard intraparotid injections, a symptom‐guided injection strategy was implemented as a second‐line escalation approach. This technique was conceptually inspired by the FTP paradigm introduced by Blumenfeld et al. in the context of botulinum toxin treatment for migraine and tension‐type headache [15], in which injection sites are selected according to patient‐reported pain localization and areas of tenderness identified on clinical examination, rather than following a fixed anatomical template.

Pain mapping was conducted during the clinical examination by eliciting first‐bite symptoms through gustatory stimulation. In keeping with the FTP principle, injection sites were determined according to the patient's pain distribution and clinically identified areas of tenderness rather than a predetermined anatomical template. While pain provocation was not standardized using a predefined citric acid concentration or volume protocol, a small quantity of lemon juice or another acidic food stimulus was administered to reproduce the characteristic symptoms. Patients were then asked to localize the areas of maximal pain intensity. The corresponding sites were marked dermographically on the skin surface and subsequently used to guide targeted BoNT‐A injections under real‐time ultrasound guidance (Figure 1). In addition to the conventional parotid injection pattern already administered during the standard protocol, an additional dose of 15–25 units of BoNT‐A was selectively administered to the targeted regions in 5‐unit aliquots per site. The specific dose within this range was individualized according to pain distribution, pain intensity, and anatomical extension of the symptomatic area, while remaining within dosage ranges previously reported in the BoNT‐A literature for salivary gland and autonomic indications. Ultrasound guidance was maintained throughout the procedure to ensure both anatomical precision and procedural safety. The overall stepwise treatment algorithm, comprising standard intraparotid injection followed by symptom‐guided FTP escalation, is summarized in Figure 2.

**FIGURE 1:**
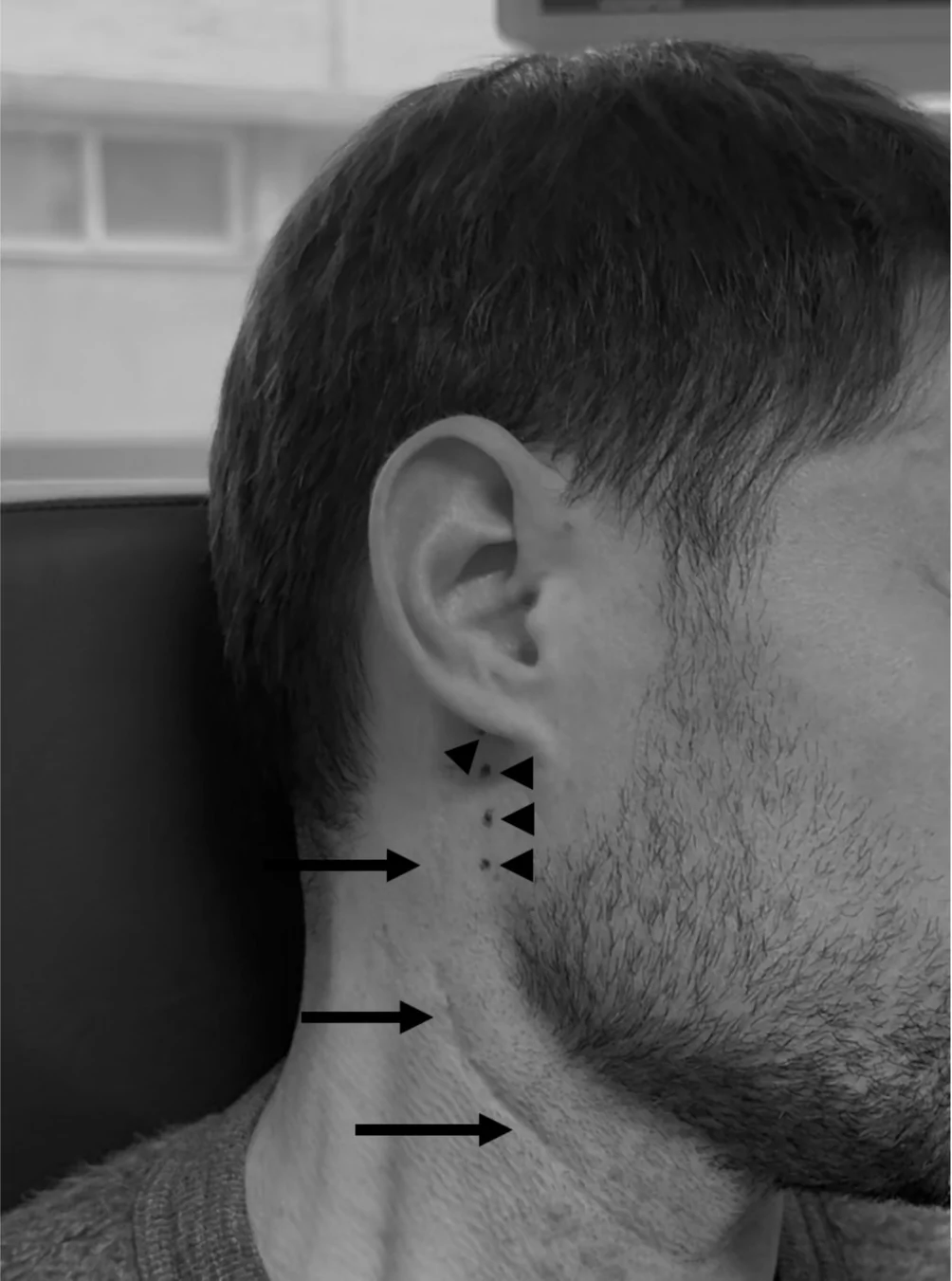
Pain mapping in First Bite Syndrome. Dermographic marking of focal pain loci identified during first‐bite provocation in a patient with postoperative First Bite Syndrome. Arrowheads indicate patient‐reported maximal pain points. Arrows indicate the cutaneous surgical scar. Marked areas were used to guide ultrasound‐assisted targeted BoNT‐A injection as part of the “Follow‐the‐Pain” (FTP) escalation protocol.

**FIGURE 2:**
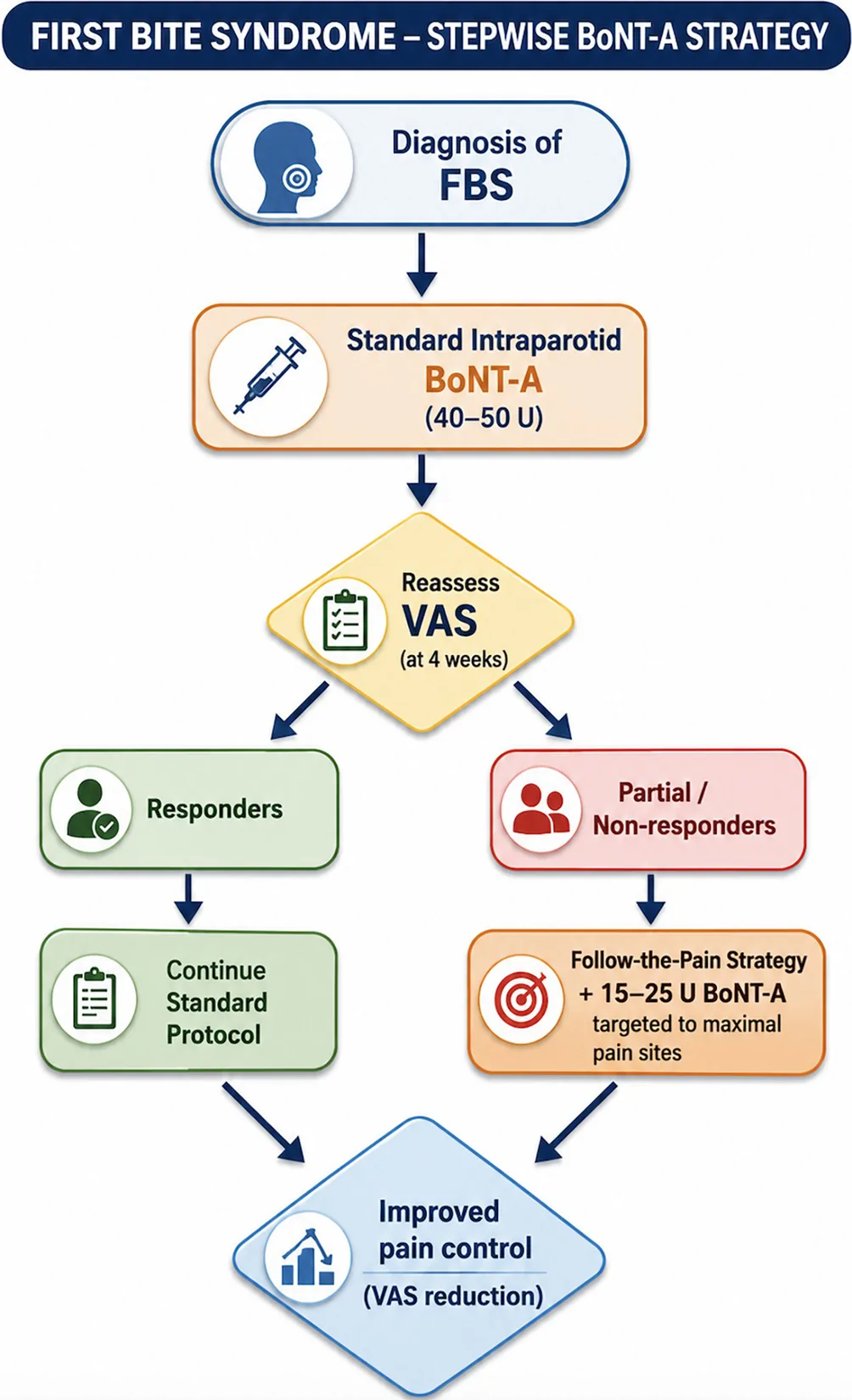
Treatment protocol. [Color figure can be viewed at wileyonlinelibrary.com]

### 2.3 Outcome Assessment

Pain intensity was assessed using a 10‐cm (100‐mm) horizontal Visual Analog Scale (VAS), anchored by the descriptors “no pain” and “worst imaginable pain.” Given the absence of validated severity categories specific to First Bite Syndrome, VAS scores were interpreted according to commonly used headache and craniofacial pain classifications derived from the International Classification of Headache Disorders (ICHD), whereby scores of 1–3 indicate mild pain, 4–6 moderate pain, 7–9 severe pain, and 10 very severe pain [18].

VAS scores were recorded before treatment (baseline) and at 4 weeks after each BoNT‐A injection session to evaluate clinical response. Treatment response was categorized as follows: marked improvement (VAS reduction ≥ 50% and final VAS ≤ 3), partial response (VAS reduction between 20% and 49%), or minimal/no response (VAS reduction < 20%). These thresholds were applied by analogy with established pain response criteria from the broader pain medicine literature and are acknowledged not to be universally standardized specifically for FBS [7, 13, 15].

All patients were monitored through a biweekly patient‐reported diary throughout the entire follow‐up period. The diary recorded both VAS pain intensity and the occurrence of meal‐related pain episodes, providing a longitudinal, granular assessment of treatment response between ambulatory visits.

Patients were reassessed 4 weeks after each treatment to evaluate clinical response and to determine eligibility for the escalation protocol.

### 2.4 Statistical Analysis

Given the exploratory nature of this case series and the limited sample size, statistical analysis was primarily descriptive. Continuous variables are presented as mean and range, whereas categorical variables are reported as counts and percentages. Changes in pain intensity were evaluated by comparing pre‐treatment and post‐treatment VAS scores at each treatment step.

## 3 Results

A total of five patients diagnosed with FBS following head and neck surgery were included in the study. The cohort consisted of three males and two females, with a mean age of 41 years (range 35–48 years). Surgical etiologies included parapharyngeal sympathetic chain schwannoma (*n* = 2), deep lobe pleomorphic adenoma of the parotid gland (*n* = 1), solitary fibrous tumor of the parapharyngeal space (*n* = 1), and parapharyngeal pleomorphic adenoma (*n* = 1). Detailed patient characteristics and treatment outcomes are summarized in Table 1.

**TABLE 1:** Patient characteristics.

Pt	Sex/Age	Site	Histology	Size (cm)	Approach	VAS pre	Standard Tx (*n*)	VAS post‐standard	FTP Tx (*n*)	VAS post‐FTP	Stable phase	Re‐injection
1	F/42	Parapharyngeal	Pleomorphic adenoma	3	C	8	2	2	—	—	Month 1–5	Yes (~month 5)
2	M/36	Parotid deep lobe	Pleomorphic adenoma	5.5	CP	8	1	6	2	2	Month 1–5	Yes (~month 5)
3	M/48	Sympathetic chain	Schwannoma	6	O	9	2	7	1	6	Month 1–5	Yes (~month 5)
4	F/44	Parapharyngeal	Solitary fibrous tumor	5.7	CP	9	8^a^	5	2	2	Month 1–5	Yes (~month 5)
5	M/35	Sympathetic chain	Schwannoma	6	C	10	2	10	2	4	Month 1–5	Yes (~month 5)

Abbreviations: C, cervical; CP, transcervical‐transparotid; FTP, “Follow‐the‐Pain” protocol; O, transoral; Pt, patient; Size, maximum diameter of lesion in centimeters; Tx, treatments.

^a^ *Patient 4* received 8 standard intraparotid injection cycles as periodic maintenance treatments, repeated approximately every 5 months when symptomatic recurrence occurred. Despite repeated standard treatment cycles, pain intensity consistently returned to a plateau of approximately VAS 5 across all cycles. The FTP strategy was introduced after this clinical plateau had been clearly established. A subsequent FTP treatment cycle confirmed the reproducibility of the additional benefit.

All patients initially underwent ultrasound‐guided intraparotid injection of BoNT‐A according to the standard protocol, consisting of 40–50 units distributed across 6–10 sites within the parotid gland. In the patient with prior parotidectomy, injections were administered into the subcutaneous tissue corresponding to the surgical bed.

Following standard intraparotid treatment, the response was heterogeneous across the cohort. One patient demonstrated marked clinical improvement, with VAS score decreasing from 8 to 2 (VAS reduction 75%), meeting criteria for marked response. One patient achieved a partial response, with VAS intensity decreasing from 9 to 5 (VAS reduction 44%). Three patients showed minimal or no clinical improvement: VAS scores decreased from 8 to 6, from 9 to 7, and from 10 to 10, respectively, representing reductions of 25%, 22%, and 0%.

Four patients, comprising the three minimal/non‐responders and the partial responder, subsequently underwent the (FTP) escalation strategy, consisting of an additional 15–25 units of BoNT‐A targeted to patient‐identified areas of maximal first‐bite pain under ultrasound guidance. Further pain reduction was observed following FTP treatment, with final VAS scores of 2 (*n* = 2), 4 (*n* = 1), and 6 (*n* = 1). Before FTP escalation, residual pain ranged from moderate to very severe intensity (VAS 5–10), whereas after FTP treatment no patient continued to report severe pain (Table 2). Response to FTP treatment was heterogeneous. Notably, patient 3 (Table 1), with a sympathetic chain schwannoma treated through a transoral approach, demonstrated a pain trajectory from VAS 9 at baseline to VAS 7 after standard intraparotid BoNT‐A treatment and VAS 6 following FTP escalation. Although the incremental benefit attributable to the FTP intervention was limited to a 1‐point reduction (VAS 7 to 6), which would not generally be considered clinically meaningful, this change was associated with a transition from severe to moderate pain according to the adopted ICHD‐based pain severity classification. This finding further illustrates the variability of treatment response and suggests a potential efficacy ceiling of the proposed strategy in selected patients.

**TABLE 2:** Pain intensity VAS scores.

Patient	Baseline	After standard injection	After FTP injection
1	Severe (8)	Mild (2)	—
2	Severe (8)	Moderate (6)	Mild (2)
3	Severe (9)	Severe (7)	Moderate (6)
4	Severe (9)	Moderate (5)	Mild (2)
5	Very severe (10)	Very severe (10)	Moderate (4)

*Note:* Changes in pain intensity following standard intraparotid botulinum toxin type A (BoNT‐A) injection and subsequent Follow‐the‐Pain (FTP) escalation protocol. Pain severity categories were defined as mild (VAS 1–3), moderate (VAS 4–6), severe (VAS 7–9), and very severe (VAS 10).

No procedure‐related complications or adverse effects associated with BoNT‐A injections were observed in any patient during the follow‐up period.

### 3.1 Longitudinal Follow‐Up

All patients were monitored through a biweekly patient‐reported diary recording VAS pain intensity and meal‐related pain occurrence throughout the follow‐up period, extended to May 2026. The diary data, available for all five patients, demonstrated:

- Near‐stable VAS scores from month 1 through month 5 post‐injection, with only minimal fluctuations.
- Persistent reduction in meal‐related pain episodes, which were reported as better tolerated throughout the stable phase, even when still present.
- A gradual decline in treatment effect beginning around the fifth month, documented by progressive VAS increase and worsening meal‐related symptoms in biweekly diary entries.

After the fifth month, all patients experienced gradual symptomatic recurrence and subsequently requested re‐injection. Repeated treatment cycles, both standard and FTP, produced comparable clinical responses, supporting the reproducibility of both protocols.

Notably, Patient 4 (Table 1) underwent eight conventional intraparotid injection cycles administered as periodic maintenance treatments, repeated approximately every 5 months when symptomatic recurrence occurred following the gradual decline of BoNT‐A effect. Treatment intervals were occasionally longer than planned due to healthcare system access constraints. Despite repeated standard treatment cycles, pain intensity consistently returned to a plateau of approximately VAS 5 across all cycles, indicating that the maximum achievable benefit from conventional intraparotid injection had been reached. The FTP strategy was introduced after this clinical plateau had been clearly established over multiple treatment cycles. Following a single FTP session, VAS decreased from 5 to 2, and a subsequent FTP treatment cycle confirmed the reproducibility of this additional benefit with a comparable pain reduction, providing evidence that the incremental improvement was attributable to the targeted injection strategy rather than to cumulative BoNT‐A exposure alone.

## 4 Discussion

FBS remains a challenging postoperative pain condition for which evidence‐based management strategies are still limited. Among the available treatment options, intraparotid injection of BoNT‐A has progressively emerged as one of the most promising minimally invasive approaches. However, despite encouraging clinical results, standardized treatment protocols and clear management strategies for patients with incomplete response remain poorly defined [9, 10, 14].

The concept of targeting botulinum toxin injections based on patient‐specific pain distribution is not entirely novel. In headache medicine, Blumenfeld et al. [15] expanded the traditional fixed‐site injection paradigm by introducing the FTP approach, in which injection sites are selected according to patient‐reported pain localization and areas of tenderness identified on clinical examination, rather than following a fixed anatomical template. This individualized strategy was subsequently incorporated as an optional component of the PREEMPT protocol for chronic migraine prophylaxis and has demonstrated clinical utility in patients with heterogeneous and variable pain distribution patterns.

In the context of First Bite Syndrome, elements of a conceptually similar approach can be identified in early reports. Ali et al. [9], in the first published description of botulinum toxin treatment for FBS, reported a case of severe, refractory symptoms treated with ultrasound‐guided injection of 75 units of BoNT‐A into the parotid gland, with particular attention directed to regions corresponding to maximal patient‐reported pain intensity, resulting in significant and durable clinical improvement. Similarly, Sims et al. [12], in a small case series of three patients treated with botulinum toxin for FBS, administered injections with partial targeting of patient‐identified painful areas, achieving substantial symptom relief in all treated cases. These observations suggest that a pain‐oriented injection strategy has been implicitly applied in prior studies of FBS. However, this approach has neither been formally conceptualized nor standardized and has not previously been evaluated within a structured, stepwise therapeutic framework specifically designed for FBS management.

The present study builds on this concept by introducing a defined, symptom‐guided FTP injection protocol and integrating it into a stepwise treatment algorithm for patients with an incomplete or refractory response to standard intraparotid BoNT‐A injection.

Available evidence on botulinum toxin treatment for FBS remains limited and heterogeneous. The systematic review by Shaikh et al. [14] identified only eight studies comprising a total of 22 patients, with no controlled trials and insufficient data for formal meta‐analysis. Most reports consist of small case series with primarily descriptive outcomes and variable follow‐up durations. Across these studies, treatment response appears variable and frequently incomplete. In a prospective series by Costales‐Marcos et al. [19], four of five patients experienced only partial improvement following standard intraparotid BoNT‐A injection, with mean VAS scores decreasing from severe pain intensity (mean VAS 8.5) to moderate pain intensity (approximately VAS 5), while one patient showed no response whatsoever. Similarly, Ghosh and Mirza [11] reported a response from severe to moderate pain intensity in three of five patients, with one non‐responder and one patient demonstrating only minimal benefit. Larger observational data reported by Handa et al. [20] further underscore this variability, with only one patient achieving complete symptom resolution following botulinum toxin treatment, while the majority experienced partial or no improvement with conventional injection techniques.

Taken together, these findings indicate that although botulinum toxin can provide clinically meaningful pain reduction in selected patients with FBS, treatment outcomes are often incomplete and unpredictable with standard intraparotid approaches. A substantial proportion of patients remain partial responders or non‐responders, even after repeated injections using conventional gland‐centered techniques. This variability strongly suggests that a uniform, anatomically fixed approach may be insufficient to address the complex and potentially spatially heterogeneous mechanisms underlying pain generation in FBS.

The present cohort mirrors this pattern. Following standard intraparotid injection, only one of five patients achieved marked pain relief meeting criteria for a clinically satisfactory response. One patient achieved partial improvement, and three patients showed minimal or no benefit—a response rate consistent with the variable outcomes documented in the broader literature [14, 19, 20].

In this context, there is a clear clinical need for more individualized therapeutic strategies capable of addressing interpatient variability in pain distribution and response to treatment. The symptom‐guided FTP technique proposed in the present study aims to address this limitation by tailoring injection sites to patient‐specific pain localization, thereby targeting focal regions of aberrant parasympathetic hypersensitivity—presented here as a biologically plausible working hypothesis consistent with current understanding of FBS pathophysiology, rather than an empirically demonstrated mechanism—that may not be adequately covered by uniform glandular infiltration. To our knowledge, no previous studies have systematically evaluated a structured, pain‐oriented injection strategy within a defined escalation framework for FBS.

The biological plausibility of this approach is supported by the current understanding of FBS pathophysiology. Surgical disruption of the cervical sympathetic pathway results in denervation hypersensitivity of parotid myoepithelial cells, consistent with Cannon's law, leading to exaggerated and painful myoepithelial contraction during gustatory stimulation [7]. The distribution and focal predominance of this hypersensitivity may vary considerably depending on the extent and anatomical level of sympathetic denervation, the surgical approach employed, and individual neuroanatomical factors. This spatial heterogeneity provides a rational mechanistic basis for individualized, symptom‐guided injection targeting. Furthermore, in addition to its well‐known inhibitory effect on acetylcholine release at cholinergic nerve terminals, BoNT‐A has been shown to exert peripheral antinociceptive effects through inhibition of CGRP release, suppression of glutamate‐mediated nociceptive signaling, and modulation of TRPV1 receptor activity at peripheral sensory terminals [13]. These mechanisms may contribute independently to the observed clinical benefit of targeted injections beyond the primary effect on myoepithelial contractility, further supporting the rationale for symptom‐guided neuromodulatory targeting in FBS.

The outcomes observed in the present series following the combined treatment strategy are presented as descriptive, informal observations and should not be interpreted as evidence of superiority over standard intraparotid injection alone, given the inherent differences in patient populations, surgical etiologies, dosing protocols, injection timing, and follow‐up durations across studies. With this caveat explicitly acknowledged, it is noted that in the series by Costales‐Marcos et al. [19] and Ghosh and Mirza [11], the majority of patients achieved only moderate residual pain intensity with standard injection alone, whereas in our cohort, the combined sequential strategy resulted in further pain intensity reduction in all four FTP‐treated patients, with heterogeneous but generally favorable outcomes. We also note that our series of five patients represents one of the largest published cohorts of FBS treated with BoNT‐A alongside these reference studies, reflecting the extreme rarity of this condition and the very limited evidence base currently available.

The heterogeneity of treatment response within our cohort warrants further consideration. In the case of sympathetic chain schwannoma treated via transoral approach (Patient 3, Table 1), the limited benefit observed following FTP escalation may reflect an efficacy ceiling related to the surgical approach and its resulting pattern of autonomic nerve disruption. Specifically, the transoral approach may produce a more diffuse or atypical sympathetic denervation compared with transcervical approaches, potentially reducing the amenability of symptoms to focal targeted injection. This hypothesis warrants further investigation in future prospective studies.

### 4.1 Treatment Durability and Re‐Injection

An important finding emerging from the extended follow‐up is the characterization of treatment durability. Biweekly diary data demonstrated near‐stable VAS scores and better‐tolerated meal‐related pain from month 1 through month 5 post‐injection across all patients, followed by a gradual decline in treatment effect. This pattern is fully consistent with the known pharmacological duration of BoNT‐A (typically 3–6 months) and with re‐injection intervals reported in analogous salivary and autonomic disorders, such as Frey syndrome and sialorrhea, where treatment cycles of 4–6 months are well established [8, 16, 17, 21].

All patients requested re‐injection after the fifth month, and repeated treatment cycles—both standard and FTP—produced comparable clinical responses, supporting the reproducibility and feasibility of long‐term cyclical management. These findings suggest that FBS management with BoNT‐A should be conceived as a long‐term, cyclical treatment program rather than a single intervention, with re‐injection anticipated at approximately five‐month intervals.

## 5 Limitations

Several limitations of this study should be acknowledged. The sample size was small (*n* = 5), reflecting both the rarity of FBS and the single‐center, retrospective design. Notwithstanding, this series represents, to our knowledge, one of the largest published cohorts of FBS treated with BoNT‐A, alongside Costales‐Marcos et al. [19] and Ghosh and Mirza [11], reflecting the extreme rarity of this condition. Nevertheless, the small sample size precludes definitive conclusions and limits generalizability.

Pain assessment relied on VAS scoring and biweekly diary recording of meal‐related pain occurrence. While the biweekly diary provided longitudinal granularity superior to a single ambulatory assessment, validated multidimensional instruments were not employed. Future prospective studies should incorporate validated patient‐reported outcome measures, including:

- OHIP‐14 (Oral Health Impact Profile) for oral health‐related quality‐of‐life assessment.
- PGIC (Patient Global Impression of Change) for global treatment satisfaction and perceived improvement.
- NRS with functional anchors for pain intensity assessment in eating‐related contexts.
- Dietary and nutritional assessment tools to capture food avoidance patterns and weight changes.

Pain provocation for FTP mapping was not standardized using a predefined citric acid concentration or volume protocol, which may limit reproducibility. Future studies should evaluate standardized approaches to pain mapping, either through a defined citric acid provocation protocol or through validated methods based on patient‐reported pain localization and clinically identified areas of tenderness to enhance methodological consistency and inter‐study comparability.

The surgical etiologies were heterogeneous; however, all patients shared a common pathophysiological background of postoperative sympathetic autonomic disruption involving the parotid region and adjacent structures, supporting the conceptual coherence of the proposed escalation strategy across different etiologies.

Finally, given the retrospective design and absence of a control arm, a contribution of cumulative BoNT‐A exposure to the observed response—particularly in Patient 4 (Table 1)—cannot be entirely excluded. However, the temporal pattern of a reproducible plateau under standard treatment followed by consistent incremental improvement, specifically after FTP escalation, confirmed across two independent FTP cycles, provides the strongest clinically available evidence that the additional benefit is attributable to the targeted injection strategy rather than to cumulative effects alone.

These findings should be interpreted as preliminary and hypothesis‐generating, rather than as definitive evidence of the superiority of the symptom‐guided approach over conventional techniques.

## 6 Clinical Implications

From a clinical perspective, the present findings support a stepwise management approach for postoperative FBS (Figure 2). Initial treatment should consist of ultrasound‐guided intraparotid BoNT‐A injection using a standardized distribution within the gland, at a dose of 40–50 units. In patients with incomplete or absent response at four‐week reassessment, early implementation of the FTP escalation strategy—consisting of additional targeted injections of 15–25 units directed to patient‐identified areas of maximal first‐bite pain intensity under ultrasound guidance—may represent a rational, safe, and potentially effective second‐line option. Clinicians should be aware that repeat injections may be required at approximately five‐month intervals and that treatment outcomes can be heterogeneous, with some patients deriving only limited additional benefit from botulinum toxin therapy and FTP escalation. The factors underlying this variability remain unclear but may include differences in surgical approach.

Future prospective studies incorporating standardized pain intensity measures, validated quality‐of‐life instruments, and longer follow‐up intervals are warranted to validate this individualized treatment model, to define optimal dosing and targeting parameters, and to identify clinical predictors of response to each treatment step.

## 7 Conclusions

Ultrasound‐guided intraparotid BoNT‐A injection represents a useful and minimally invasive first‐line treatment option for postoperative FBS [9, 10, 11, 12, 19, 20]. However, the present series confirms that a substantial proportion of patients, consistent with findings from prior studies [14, 19, 20], do not achieve satisfactory pain control with standard intraparotid injection alone, highlighting the need for structured escalation strategies in clinical practice.

The symptom‐guided FTP technique, formally introduced in the present study as a defined escalation protocol for FBS, was applied in four of five patients and resulted in further pain intensity reduction in all treated cases, with heterogeneous outcomes. Two patients achieved marked pain reduction (VAS 2), one achieved moderate improvement (VAS 4), and one showed limited additional benefit (VAS 6), reflecting the variability of treatment response.

All patients maintained near‐stable VAS scores and better‐tolerated meal‐related pain until approximately the fifth month post‐injection, as documented by biweekly diary monitoring, followed by gradual symptomatic recurrence requiring re‐injection. Repeated treatment cycles produced comparable responses, supporting the feasibility of long‐term cyclical management with re‐injection anticipated at approximately five‐month intervals.

The proposed approach is conceptually grounded in the established FTP paradigm of Blumenfeld et al. [15] and is mechanistically supported by the known pathophysiology of FBS and the peripheral antinociceptive properties of BoNT‐A [13].

By targeting focal regions of aberrant parasympathetic hypersensitivity—presented as a biologically plausible working hypothesis rather than an empirically demonstrated mechanism—that may not be adequately addressed by uniform glandular infiltration, the technique offers a rational, individualized, and potentially effective escalation option for patients with refractory or partially controlled symptoms.

Integration of this symptom‐guided strategy into a stepwise management algorithm, with the expectation of long‐term cyclical re‐injection at approximately five‐month intervals, may improve therapeutic outcomes in this challenging condition. Prospective studies with larger cohorts and standardized outcome measures are warranted to confirm and extend these preliminary findings.

## Funding

The authors have nothing to report.

## Conflicts of Interest

The authors declare no conflicts of interest.

## Data Availability

The data that support the findings of this study are available from the corresponding author upon reasonable request.
